# Relational Processes in Ayahuasca Groups of Palestinians and Israelis

**DOI:** 10.3389/fphar.2021.607529

**Published:** 2021-05-19

**Authors:** Leor Roseman, Yiftach Ron, Antwan Saca, Natalie Ginsberg, Lisa Luan, Nadeem Karkabi, Rick Doblin, Robin Carhart-Harris

**Affiliations:** ^1^ Centre for Psychedelic Research, Imperial College London, London, United Kingdom; ^2^ Faculty of Social Sciences, Hebrew University, Jerusalem, Israel; ^3^ School of Creative Arts Therapies, Kibbutzim College, Tel Aviv, Israel; ^4^ Multidisciplinary Association for Psychedelic Studies (MAPS), Santa Cruz, CA, United States; ^5^ Anthropology Department, University of Haifa, Haifa, Israel

**Keywords:** ritual, spirituality, relationship, intergroup contact, ayahuasca, psychedelic, peacebuilding

## Abstract

Psychedelics are used in many group contexts. However, most phenomenological research on psychedelics is focused on personal experiences. This paper presents a phenomenological investigation centered on intersubjective and intercultural relational processes, exploring how an intercultural context affects both the group and individual process. Through 31 in-depth interviews, ceremonies in which Palestinians and Israelis drink ayahuasca together have been investigated. The overarching question guiding this inquiry was how psychedelics might contribute to processes of peacebuilding, and in particular how an intercultural context, embedded in a protracted conflict, would affect the group’s psychedelic process in a relational sense. Analysis of the interviews was based on grounded theory. Three relational themes about multilocal participatory events which occurred during ayahuasca rituals have emerged from the interviews: 1) *Unity-Based Connection* – collective events in which a feeling of unity and ‘oneness’ is experienced, whereby participants related to each other based upon a sense of shared humanity, and other social identities seemed to dissolve (such as national and religious identities). 2) *Recognition and Difference-Based Connection –* events where a strong connection was made to the other culture. These events occurred through the expression of the other culture or religion through music or prayers, which resulted in feelings of awe and reverence 3) *Conflict-related revelations* – events where participants revisited personal or historical traumatic elements related to the conflict, usually through visions. These events were *triggered* by the presence of ‘the Other,’ and there was a political undertone in those personal visions. This inquiry has revealed that psychedelic ceremonies have the potential to contribute to peacebuilding. This can happen not just by ‘dissolution of identities,’ but also by providing a space in which shared spiritual experiences can emerge from intercultural and interfaith exchanges. Furthermore, in many cases, personal revelations were related to the larger political reality and the history of the conflict. Such processes can elucidate the relationship between personal psychological mental states and the larger sociopolitical context.

## Introduction

“*Those who experience do not participate in the world. For the experience is “in them” and not between them and the world*.”

-Martin Buber, *I and Thou (1923*, p 55)

Although the Israeli-Palestinian conflict is rooted in competition over material resources and political or territorial control, numerous studies emphasize the role identities and narratives play in preserving the conflict (Hammack, 2008; Hammack, 2009; Bekerman and Zembylas, 2011; Hammack, 2011), in denying the legitimacy of the other (Bar-On and Adwan, 2006), and in structuring the reality of the conflict as a “zero-sum game” (Bar-Tal and Salomon, 2006; Klar and Baram, 2016). These opposing national and religious group identities and narratives associate the conflict with a heavy load of sentiments, including fear, disparagement, blame and grudge (Bar-Tal and Salomon, 2006) accompanied by a socio-psychological repertoire of attitudes, objectives and beliefs regarding the causes of the conflicts and its course and regarding the adversary (Bar-Tal et al., 2010; Bar-Tal and Halperin, 2011; Bar-Tal and Halperin, 2013). By doing so, these conflict-based group identities contribute to the deepening and perpetuation of the culture of conflict (Bar-Tal and Salomon, 2006; Bar-Tal, 2007). Both Palestinian and Israeli collective identities are inextricably constructed in relation to one another in terms of conflict.

In the context of these processes, encounters aimed at reconciliation and other forms of intergroup contact - mostly between Jewish and Palestinian citizens of Israel - are commonly employed to alter intergroup attitudes and improve relations between the two sides (Abu-Nimer, 2004; Ron et al., 2010). Maoz (2004) defines two main characteristics of the sociopolitical context of the conflict between Jews and Palestinians in Israel, which are particularly relevant to the encounter between the two groups: 1. Relationships of conflict and aggression alongside coexistence and cooperation. 2. Inequality in which Jews have greater access to resources and influence over the culture, religion and language of the State. Thus, like other contact interventions conducted in settings of ethnopolitical conflicts, intergroup encounters between Jewish Israelis and Palestinians constitute a paradoxical project that aims to bring about open dialogue, equality and cooperation between two groups embedded in a deep-rooted reality of protracted conflict and asymmetry (Suleiman, 2004; Maoz, 2011; Ron and Maoz, 2013a; Ron and Maoz, 2013b).

This study seeks to explore the potential role of the psychoactive brew ayahuasca in settings of intergroup contact to shift the awareness and attitudes related to the relations between groups embedded in ethnopolitical conflicts, including long-term disputes between Jewish Israelis and Palestinians. By doing so, the present study also seeks to 1) understand how psychedelics can affect relational and group-participatory processes and 2) expand knowledge on dynamics between these two groups by focusing on their interactions in a psychedelic group setting.

Classic psychedelics are a family of drugs whose central mode of action is through the 2A receptor of the serotonergic system (Nichols, 2016). Their effect is known to be context-dependent (Carhart-Harris et al., 2017; Hartogsohn, 2017; Hartogsohn, 2020), and they are known to produce a myriad of intense experiences (Harner, 1973; Grof, 1975/2016; Shanon, 2002), including so-called mystical-type and ego-dissolution experiences (Pahnke and Richards, 1966; Griffiths et al., 2008; Nour et al., 2016), emotional breakthrough, release or catharsis and psychological processing of traumatic events (Frederking, 1955; Riba et al., 2006; Gasser et al., 2014; Belser et al., 2017; Roseman et al., 2019), challenging high-anxiety experiences (Carbonaro et al., 2016), prophetic-type experiences (Strassman, 2014), visionary and hallucinatory experiences (Knauer and Maloney, 1913; Klüver, 1926; Reichel-Dolmatoff, 1975; Luna and Amaringo, 1991/1999; Shanon, 2002; Roseman, 2019), and experiences of social connection (Dolder et al., 2016; Pokorny et al., 2017; Apud, 2020b; Forstmann et al., 2020; Kettner et al., 2021). The context-dependent quality of psychedelics has led them to be referred as ‘non-specific amplifiers’ (Grof, 1975/2016) – meaning they intensify whatever is in the vicinity of the experiencer, whether good or bad or intrinsically (e.g., in their own mind) or extrinsically located (i.e., in their immediate environment). It has been highlighted that serotonergic 2A receptors are modulated by stress (‘upregulated’) and important for promoting plasticity – which accounts for the ability of psychedelics to enhance sensitivity to context (Carhart-Harris and Nutt, 2017). Psychedelics and psychedelic therapy more specifically, could be perceived as a quintessentially biopsychosocial intervention (George and Engel, 1980; Winkelman, 2010), and the biopsychosocial model is very relevant to the present investigation.

The psychedelic investigated in this study is commonly known by the Quechua-language name “ayahuasca”, an Amazonian brew whose main two pharmacologically functionally relevant components are typically two plant-derived ingredients: DMT – a classic tryptamine psychedelic – and a mix of MAO inhibitors which prevents the breakdown of DMT when it is consumed orally (Rivier and Lindgren, 1972). Ayahuasca has been cultivated, prepared and consumed for centuries in some indigenous and mestizo (a sociocultural hybrid of indigenous and Spanish cultures) Amazonian cultures, a practice which has been widely diffused and diversified across the amazon at the time of the rubber boom (Gow, 1994; Brabec de Mori, 2011). In these practices, the facilitator, who is often called a Shaman^1^, uses it to ‘heal’ different maladies or as part of a spiritual practice; by serving it to participants, and/or by personally consuming it themselves (Dobkin de Rios, 1973; Luna, 2011). In many cases, the experiences observed in such settings fit the cosmology and sociocultural structure of the indigenous or mestizo people (Langdon, 1979; Dobkin De Rios, 1984). Reichel-Dolmatoff (1972) suggested that while simple geometric imagery is cultural independent and might reflect the brain structure of the visual cortex, complex imagery is culture dependent. A notable interaction between Amazonian culture and the ayahuasca experience is centered on animism. Within an animistic ontology, the world is inhabited by anthropomorphic spirits, and ayahuasca facilitates communication with them (Luna, 2011). For example, the shaman can have an out-of-body experience - with a sensation of flight - in which they travel into the spiritual realms and encounter the spirits, demons, and deities which inhabit the jungle, as well as snakes and jaguars (Harner, 1973).

Ayahuasca expanded globally via three main vehicles (Tupper, 2009; Labate and Jungaberle, 2011). The first is via Brazilian syncretic churches who use ayahuasca with a communal-religious intention, while borrowing from different spiritual and religious traditions such as Christianity, African rituals and Amazonian traditions (Soibelman, 1995; Labate and Macrae, 2016). Santo Daime and Uniao do Vegetal (UDV) are the largest of such traditions. The second vehicle is via shamanic tourism (Fotiou, 2010b; Fotiou, 2020) in which mainly western seekers travel to the Amazon (or other locations in the world in which ayahuasca is legal or decriminalized), and partake in ayahuasca ceremonies seeking psycho-spiritual growth (Winkelman, 2005). Shamanic tourists and seekers are in a quest to ‘heal’ from what they perceive as the maladies of western culture, and criticism toward western culture is at the root of this pursuit for healing by the non-western ‘other’ (Gearin, 2016b; Fotiou, 2020). In these contexts, a group gathers for a couple of days to few weeks, but usually does not stay in touch afterward. These ceremonies, programs, or retreats tend to combine religious and therapeutic elements (Fotiou, 2020) and sometimes mix traditional ayahuasca shamanism with western theory and styles of psychotherapy (Loizaga-Velder and Verres, 2014; Marcus and Fotiou, 2019). As the western travelers seek psychospiritual personal healing, the interpretative framework is sometimes psychologized, and the practice is sanitized from what is considered by westerners as ‘darker’ aspects of Amazonian shamanism, like sorcery (Fotiou, 2010a; Brabec De Mori, 2014). The third line of spread, which is usually more *underground*, is worldwide, local ayahuasca circles typically led by local facilitators, sometimes referred to as neoshamans (Scuro and Rodd, 2015). The facilitators blend indigenous practices with some forms of Western therapeutic practices, *new-age*, religious elements, and other spiritual traditions and practices based on their own identity, or local to their countries. In many cases, people tend to return to the same group, and communities are formed around these ayahuasca ceremonies (Hanegraaff, 2011; Rodd, 2018). While these groups often evolve into communities, the language they use and intentions for taking ayahuasca are still centered on therapeutic ends and personal processes (Gearin, 2015; Rodd, 2018). The interviewees in this study belong to these local neoshamanic groups – mixing a number of traditions within their practice.

The field of psychedelic research has been expanding quickly, with most research focused on these compounds’ therapeutic potential in private settings (Mithoefer et al., 2016; Carhart-Harris and Goodwin, 2017; Nichols et al., 2017; Palhano-Fontes et al., 2019) and their neurobiological action (Riba et al., 2002; Preller et al., 2018; Carhart-Harris and Friston, 2019; Timmermann et al., 2019). However, psychedelics are widely used in other contexts, and are often used in groups. In many indigenous and mestizo cultures the ingestion of psychedelics was not just for individual treatment, but for socially constructive purposes embodied in public rituals (La Barre, 1938/1975; Furst, 1972; Hofmann and Schultes, 1979; Dobkin De Rios, 1984; Rätsch, 2005) where they supported the creation of social identity (Reichel-Dolmatoff, 1978; Taussig, 1987; Andritzky, 1989; Winkelman, 2010; Guerra-Doce, 2015; Langdon, 2016), and supported intercultural and interethnic exchange as well (Gow, 1994; Brabec de Mori, 2011; Langdon, 2013; Fotiou, 2020). While acknowledging that ayahuasca shamanism was designed and diffused under colonial pressure (Taussig, 1987; Gow, 1994; Brabec de Mori, 2011)– and that what is perceived as traditional or authentic is the product of a complex feedback loop between indigenous people, academics and tourists (Langdon, 2013; Saéz, 2014; Fotiou, 2020) - the creation of social identities through the use of ayahuasca is considered by some as both personally and politically important (Langdon, 2016). Some argue that ayahuasca shamanism is supporting dialogue between indigenous people and western society which is beyond the unidirectional forces of colonization, extraction and appropriation, though it is still impacted by such colonial forces (Langdon, 2013; Fotiou, 2020). The history of ayahuasca’s diffusion within and without the Amazon, and between urban and rural areas, suggests that ayahuasca can be perceived as a sociopolitical bridge – a potential which is crucial for the current investigation.

Despite the prevalence of group-use of psychedelics and their known influence on the construction of social identities, there is little research on the *phenomenology* of interpersonal and intercultural processes. Previous qualitative phenomenological research focused on neoshamanic practices of ayahuasca has shown experiential themes aligned with other psychedelic research. For example, participants can experience visual imagery, awe and reverence, sense of connection, insights, emotional release, feeling the sense of God or a ‘higher power,’ traveling through time and space, review of the past, communicating with spirits and other beings, death and near-death experiences, reliving trauma, experiencing ‘demonic’ forces,’ a strong sense of peace, embodied experiences, trance or possession experiences, and metacognitive awareness in which one can ‘observe’ his own mental processes (Kjellgren et al., 2009; Trichter et al., 2009; Fernández and Fábregas, 2014; Loizaga-Velder and Verres, 2014; Apud, 2015; Apud, 2020a). Yet, in addition to the above personal experiences, there are important participatory and relational elements in ayahuasca ceremonies that deserve greater attention from researchers. Several studies have reported psychedelic induced group emotional synchrony, *communitas,* or other relational processes, whether in rituals (La Barre, 1938/1975; Gearin, 2016a; Kettner et al., 2021), festivals (Saldanha, 2007; St John, 2009; Winkelman, 2015; Forstmann et al., 2020), or group therapy (Trope et al., 2019). Within some indigenous shamanic traditions, healing is pursued through the relational act of singing. The songs sang by the shaman are sometimes considered a ‘sonic substance’ which remains in the body of the patient (Brabec De Mori, 2015). Furthermore, songs are used to exert cultural influence on the visions (Langdon, 1979). Furthermore, research into neoshamnic groups and shamanic tourism reveals that the ayahuasca-induced ‘purge’ has relational elements - including social, emotional and interpersonal process - in which the gut is conceived as the “mediator between nature and culture” (Fotiou and Gearin, 2019). Yet, while some relational elements are known, qualitative phenomenological descriptions of such effects are still limited.

A considerable part of modern psychedelic research – and especially its psychopharmacological investigation - remains culturally and intellectually influenced by transpersonal theory (Grof, 1998; Ferrer, 2017), perennial philosophy (Huxley, 1945; Baier, 2017), and new-age culture (Heelas, 1996). These frameworks have limitations. Transpersonal psychology – the so-called ‘spiritual fourth arm’ of psychology – is biased in its focus toward intrasubjective (‘inner’) experience, and claims that such experiences are ‘perennial’ or universal (Huxley, 1945; Maslow, 1964; Grof, 1975/2016). This is similar to the wider *inner-spirituality* which is the center of new-age – a spirituality which is not dependent on organized religions and is gained through personal experiences (Heelas et al., 2005). Emphasis on the *inner* quality of spiritual experiences began with modernity and the detachment from organized religions (Rothberg, 1993), but became widely popular after William James’ Varieties of Religious Experience (1902), Carl Jung’s collective work (Jung, 1928/1970), and Abraham Maslow’s research on *peak* experiences (Maslow, 1959). Furthermore, the individualistic focus on inner experiences within psychedelic therapy relates to the humanistic origins of transpersonal psychology, and to the need to associate spiritual processes within psychological ones. Maslow, who was arguably the original humanistic psychologist, later became a key initiator of transpersonal psychology together with Stanislav Grof. Yet even Maslow himself – who popularized ‘peak experiences’ – warned that “some people run the danger of turning away from the world and from other people to search for anything that will trigger peak experiences. This type of person represents the *mystic gone wild*” (Krippner, 1972). Investigating the relationship between transpersonal processes and sociopolitical and interpersonal reality can prevent spiritual bypassing (Masters, 2010) and support the ‘mystic’ in her ‘return’ back to the world.

Concerns with the over-emphasis of inner-spirituality are described extensively in new-age research (Heelas, 2009; Simchai, 2009). In short, while new-age culture sees itself as a counter-culture, it can actually reinforce dominant cultural trends of individualism, something which has been observed in some neoshamanic ayahuasca circles as well (Gearin, 2015; Rodd, 2018; Apud, 2020a). For example, some Australian ayahuasca practitioners believe that “The primacy of the individual is reflected in the idea that social transformation is only possible after the individual transforms their consciousness” (Rodd, 2018). This is aligned with the Israeli new-age ethos (Simchai, 2009), including the ethos of the population observed in this study. Within this ethos, ‘peace starts from within’ and social change happens through personal change which ‘ripples’ out, or by achieving a ‘critical mass’ of individuals who have gone through personal transformation. Important to note, that such new-age ideology in Israel can sometimes support political amotivation, and lead to ‘identity blindness’ which can serve hegemonic power relations (Simchai, 2014; Simchai and Keshet, 2016).

The drawbacks of inner-spirituality are also summarized well in Ferrer’s (2002) critique and revisioning of Transpersonal theory. Some of the problems that Ferrer points to are: 1) Intrasubjective reductionism - in which one is motivated to search for meaning *within* oneself, and in doing so, one becomes separated from other social contextual factors. This is similar to critiques of traditional forms of psychiatry (Alexander, 2008; Hari, 2019) and psychology (Bistoen et al., 2014) which claim that by a narrow focus on biological or psychological mechanisms, respectively, they conceal social factors of mental health concerns, and therefore prevent addressing them. Within the framework of inner-spirituality, personal healing requires *releasing blame* for things outside of an individual’s control, *accepting* things as they are, and seeking remedy *within* by changing the individual response to external forces. This can inhibit social change. Furthermore, this can lead to pressure on subjectivity by creating anxiety within people who internalize the same blame when a remedy is difficult to achieve due to pressure from unacknowledged *outer* social constrains (Rothberg, 1993). 2) Spiritual Narcissism (Lasch, 1979/2018), a concept well documented in many religious traditions (May, 1982), describes when spiritual experiences are used for self-aggrandizement; people believe they are superior to others because of their extensive spiritual experiences. In this sense, a spiritual ego-inflation can serve as a *spiritual bypass* to avoid engaging with other aspects of one’s life (Masters, 2010) 3) Integrative arrestment describes the challenge of integrating spiritual experiences into everyday life. Over-emphasis on *experiences* can conceal spirituality’s relation to everyday life*:* “regardless of the quantity, spiritual experiences do not produce a spiritual life” (Ferrer, 2002)*.* This can lead people to have a ‘collection of experiences’ they consider spiritual highs (Bauman, 1998), yet these experiences fail to result in a truly transformative change, and fail to address problems such as social isolation and injustice (Carrette and King, 2004). In these cases, a person can become dependent on seeking *inner* experiences without attempting to change their *outer* relational life accordingly. It is suggested that this can become worse when spirituality is commodified (Ward, 2008), which is the current situation for ayahuasca (Tupper, 2009) and other psychedelics.

Recent trends within transpersonal psychology and western spirituality have begun to challenge some of the assumptions of transpersonal psychology, bringing in more of a focus on relational and participatory phenomena (Ferrer, 2002; Heelas et al., 2005; Gearin, 2016a). Ferrer suggested that western spirituality is going through a ‘participatory turn’ and that in this ‘participatory turn’ spiritual processes should be reconsidered as *multilocal participatory events*: 1) Multilocal: the event incorporates elements which are intrasubjective, relational, communal, based on collective identities, and related to places. 2) Participatory: the person is not just a passive experiencer but is active in the co-creation of the spiritual event. 3) Events: unlike the word ‘experience’ which implies a subject who experiences, and a phenomenon/world that is experienced, the word ‘event’ adds the broader context into account. Ferrer suggested that a participatory focus of spiritual events, can support the investigation and utilization of these states in interfaith and intercultural contexts. Throughout the paper multilocality and participatory processes will be emphasized – this is done not to negate intrasubjective experiences, but to emphasis relational elements which are usually neglected in psychedelic research.

One of the relational processes observed in psychedelic ceremonies and festivals is communitas (Tramacchi, 2000; St John, 2008; Kettner et al., 2021). Victor and Edith Turner describe communitas as a temporary state in which social structure and power dynamics dissolve (Turner, 1969/2017; Turner, 2012). Communitas was described by Victor Turner while investigating African rites of passage rituals in which moments of *liminality* occur (Van Gennep, 1909/2013). The ritual process creates a temporary liminal space in which relations and identities of ordinary social life become loosened, inverted, or rendered unimportant. Therefore, it can lead to relationships between group members that are not based on the regular social hierarchy and power dynamics, but instead based on shared humanity; Turner defines communitas as an anti-structure. Communitas is usually a moment of ‘collective joy,’ togetherness and common humanity (Turner, 2012), and many times people describe it as if ‘there was magic in the air.’ While some rituals are intentionally designed to create communitas, people can also unexpectedly experience communitas in other places including festivals (St John, 2008), music concerts (Diederich-Hirsh, 2010), and in moments of natural disasters (Jencson, 2001; Turner, 2012). Communitas, and other similar communal and relational processes, have been observed in some anthropological studies of psychedelics (La Barre, 1938/1975; Tramacchi, 2000; Panneck, 2014; Gearin, 2016a) including during ayahuasca neoshamanic ceremonies where “the body and the senses in the techniques by which ecstatic healing and wisdom are achieved [can] include forms of relational personhood in which the “I” of the drinker may incorporate other people, spirits, and psychic objects that relate to the actions of other people” (Gearin, 2016a). Furthermore, a large online quantitative study showed that acute communitas was high during psychedelic ceremonies, and that it mediated long term changes in well-being and increased social-connectedness (Kettner et al., 2021). Psychedelics can amplify elements pertaining to the ritual process, (as they do the therapeutic process), and make communitas more accessible to the group. More broadly, social and anthropological studies have largely concluded that rituals can increase group cohesion (Durkheim, 1912/2008; Whitehouse et al., 2014). This idea dates back to at least the 14th-century Muslim scholar, Ibn Khaldun, who studied *asabiyah* (solidarity/social cohesion) and suggested that *asabiyah* is stronger in small groups than in urban environments, and that rituals preserve it (Khaldun, 1377/2015). Rituals with ayahuasca and other psychedelic and shamanic practices can also generate strong social cohesion (Winkelman, 2010; Panneck, 2014), which helped inspire this study.

As identities could potentially play a significant role in the Israeli-Palestinian conflict (Hammack, 2008; Hammack, 2009; Bekerman and Zembylas, 2011; Hammack, 2011), shifts of identities and the relation between them can play a significant role in peacebuilding. Peacebuilding, defined here, is not just an achieved state of harmony, but striving toward political liberation as well (Abu-Nimer et al., 2007). We hypothesized that communitas in the investigated context could provide a momentary dissolution of separate identities, and create an opportunity for people to relate to each other through shared humanity and potentially start to build trust. However, as the cultural context is known to affect the psychedelic experience (La Barre, 1938/1975; Wallace, 1959; Hartogsohn, 2017; Hartogsohn, 2020) we also expected that some of the observed events and experiences would be related to the conflict itself, to history, and to local identities. Therefore, the rituals may provide not just an opportunity to celebrate ‘shared humanity,’ but also to challenge one’s narrative by encountering ‘the Other’ (Ron and Maoz, 2013a). The manifestations of both ‘unity’ and ‘diversity’ (Abu-Nimer et al., 2007) are theoretically plausible under the influence of psychedelics, which is what we set to investigate. We interviewed people who have drunk ayahuasca in mixed groups of Israelis and Palestinians. We studied these groups using qualitative methods, applied to in-depth interviews. This paper focuses on relational processes occurring *during* ayahuasca ceremonies. A separate paper will discuss changes that occurred *after* these ceremonies.

## Materials and Methods

Ethics approval was granted by Joint Research Compliance Office at Imperial College London and the Imperial College Research Ethics Committee (ICREC reference 18IC4346).

### Research Population and Ceremonies

Thirty-one participants were interviewed: 13 Arab Palestinians (five women, and eight men; seven from Christian background, and six from Muslim background; nine have Israeli citizenship and four live under occupation in the West Bank) and 18 Jewish Israelis (eight women, nine men, and one non-binary person. Ages ranged between 28 and 59. All interviewees attended ayahuasca ceremonies in which participants were both Israelis and Palestinians. Interviewees belong to five different ayahuasca groups. The main facilitators of these groups were two Jewish-Israeli men, one Jewish-Israeli woman, one Arab-Palestinian man, and one European man. Most of the interviewees were affiliated to one of these groups, but some were affiliated with multiple groups, or occasionally joining ceremonies of other groups. Not all ayahuasca groups in Israel are inclusive, and interviewees were chosen based on their history in participating in such inclusive ceremonies. Most interviewees were substantially experienced with ayahuasca. The main reported intention for participation in ceremonies was personal psychospiritual growth; none of the ceremonies was conducted with reconciliation or peacebuilding as a primary stated intention. Participants were from different political backgrounds when they first joined the ceremonies.

Ceremonies ranged from 6 to 40 participants, though most of the ceremonies included around 20 participants. Some of the groups were active for many years, and many of the participants returned to the same group and participated in ceremonies a few times a year. In order to trust newcomers, groups are formed in a ‘friend-brings-friend’ style. Music plays a crucial part in the ceremonies and is typically eclectic. Facilitators and other musicians lead the ceremony with music; there are moments in which the group sings together; and sometimes participants can share their own music or prayers as well. The ceremony participants’ interpretive framework was aligned with new-age culture and inner-spirituality (Heelas, 1996; Heelas, 2009; Simchai, 2009). Ceremonies were influenced by South American ayahuasca traditions and neoshamanic culture (Labate and Jungaberle, 2011), and also had Jewish, Muslim, Christian, Buddhist and Hindu elements. Most ceremonies were at night, and participants drank two to three cups of ayahuasca throughout the evening. The length of ceremonies ranged from approximately 4 to 8 h. Some ceremonies were part of a retreat in which multiple ceremonies took place. ‘Sharing-circles’ were common both before and after the ceremonies. Participants pay to take part in ceremonies and prices per ceremony varied from around $50 to $200.

Important to note that Palestinian participants were a minority in the ceremonies in a number of levels: 1) The number of Palestinian participants in most of the observed ceremonies was no bigger than 20%; 2) The dominant language of the groups was Hebrew; 3) Jewish-Israelis were more acquainted with new-age culture and therefore had a larger (sub) cultural capital (Bourdieu, 1979/1984; Thornton, 1996); 4) Facilitators, musicians and helpers were mainly Jewish-Israelis (except one group which was led by Arab-Palestinian facilitator), and most Jewish-Israelis participants had more experience with ayahuasca). This created some power imbalance in which Arab-Palestinians - which are already a minority - relied on the support and guidance of Jewish-Israelis.

### In-Depth Interviews

The analysis presented here is based mainly on in-depth interviews of Jewish-Israelis and Arab-Palestinians who participated in ayahuasca ceremonies together. Interviews were conducted at a location chosen by the interviewee, usually their home or workplace. The interviews were semi-structured and included four parts: 1) Background, 2) questions about general ayahuasca use, 3) questions about ayahuasca in an Israeli-Palestinian context, and 4) a dialogue – using a possible bank of challenging and stimulating questions – in which the interviewer was able to express his/her thoughts as well. LR conducted 20 interviews, AS conducted seven interviews, and NG conducted four interviews. Interviews were conducted in Hebrew, Arabic, or English. Each interview lasted from one to two and a half hours. All interviews were recorded and transcribed. All Arabic interviews and nine Hebrew interviews were translated into English to allow different authors to read the transcripts.

### Participant Observations

Two participant observations (i.e., during two ceremonies) were conducted by LR. The first was with a group of mainly Palestinians for the period of one entire ceremony and its integration the day after, and the second with a group of mainly Jewish participants for two ceremonies (in two consecutive days) and their integration the day after. Field notes were taken only during the second observation. These participant observations were done to collect secondary and complementary information by getting closer to the interviewees’ world, culture and ritual process, and to help verify the reliability of the content of the interviews.

### Data Analysis

The analysis was based on the grounded theory approach (Glaser and Strauss, 1967/2017). This approach emphasizes hypothesis-free bottom-up generation of concepts and themes. In line with this approach, several stages of analysis were undertaken (Berg, 1988/2004; Strauss and Corbin, 1990; Shkedi, 2003; Shkedi, 2019). The first phase included a thematic analysis of the interviews, which revealed thematic categories. Through a process of reading and re-reading the interviews, the number of categories was reduced by combining similar categories and focusing on those that emerged as most relevant. These categories were scrutinized again for centrality (repeated appearances across interviews and observations), for the connections between them, and for their relevance to the study and the questions it addresses. Narralizer was used to organize and code the interviews (Shkedi and Shkedi, 2005). Narralizer is a simple software for organizing and structuring qualitative data, and no automatic analysis was conducted.

See the Supplementary Material for more details on Methods, including sections on Initial Inquiry; Semi-structured interview; Coding; Ayahuasca Circles; Facilitators and Groups; and Interviewees’ background.

## Results

The identified relational themes are 1) *Unity-Based Connection:* a connection based on similarities; 2) *Recognition and Difference-Based Connection:* a connection to and recognition of ‘the Other’; and 3) *Conflict-related Revelations*: personal or historical revelations (mostly visionary, but sometimes emotional or cognitive) related to the Israeli-Palestinian conflict.

## Unity-Based Connection

During the ceremonies, a common event that occurred to individuals, or to the group, was to relate to each other beyond local collective identities (Palestinian, Israeli, Jewish, Muslim, Christian, etc.), and based on shared *universal* similarities (18/31). Interviewees described this as moments of ‘unity,’ ‘oneness,’ or as a strong sense of ‘togetherness.’

There is, however, a multilocality to these events, in that they occur both in the individual and between them in terms of group relations. Moreover, they resemble in their description many other ritual processes that have been reported without the use of psychedelics (Turner, 1969/2017), which are known to happen with psychedelics as well (Kettner et al., 2021). Descriptions of this theme were relatively homogenous across interviewees, and this theme is well-documented within psychedelic research and culture. Therefore, we decided not to expand on it compared to the other themes, and we chose only a few examples.

*Jewish Israeli woman:* “[We] really experienced this place in which the connection is not Israel-Palestinian, it is human, the human tribe.”

*Arab-Palestinian woman from Israel:* “You reach that point when you don’t see this as Jewish or Arabic … there’s nothing, no language, no religion, no gender, nothing.”

*Arab-Palestinian man from West-Bank:* “I now enjoy doing it with Palestinians and Israelis because when the journey starts, everything just goes into a state of Unity, to the energy that exists between us. We stop viewing each other as you’re Israeli, or you are Palestinian. We stop viewing each other as male or female. We don’t view Muslim, Christian, it all melts, like melts down, and dissolves through the journey.”

*Jewish-Israeli woman:* “as soon as people take the medicine and they open up to a more universal consciousness they are operating more on a soul level as opposed to an ego personality level. So as soon as they go up to their soul level the boundaries dissolve and you go back to I'm human and were all one and were connected and the heart opens. And you're just present for the other people and you're connected on a deep deep level. All the other stuff is just the illusion of separation and it's like ego and you just get past that briefly.”

## Recognition and Difference-Based Connection

Though most of the interviewees believe in the importance of universal connection beyond identity, most of them also reported a connection to ‘the Other’ based on ‘non-universal’ local identities (21/31). In most cases, this was an important transformative moment during the ceremony, which, for instance, was expressed by a Jewish-Israeli man as: “when you finally said this one thing that you wanted to say, 3 h into a discussion, but you were too afraid to say it, and if it’s going well, it’s like something is missing and then suddenly nothing is missing and there is this sense of peace … Suddenly you breathe easier.” As the ceremonies’ interpretive framework is considered ‘apolitical’ and ‘universal’ (beyond national and political identities) – much in alignment with the Israeli *new-age* ethos (Simchai, 2009) - it can exclude conflictual political discourse around national identities. This is a phenomenon that was observed in many interfaith dialogue groups which are searching for spiritual and religious connection and harmony, in an ‘apolitical’ manner, and therefore marginalize discussions on the need for political liberation (Abu-Nimer et al., 2007). When political reality ‘leaks’ into the ceremony through these moments of recognition, it is sometimes associated with a feeling of relief or awe, as what was consciously or unconsciously excluded, is now included. Awe is associated with the need to accommodate novel information which overwhelms existing mental structures (Keltner and Haidt, 2003), and therefore likely to be felt in moments of transformative recognition.

Most of the events shared by interviewees were moments in which Jewish-Israelis recognize and connect to elements of Arab-Palestinian culture and history. Power balance was asymmetric both outside of the ceremony, and in the ceremony. Within the ceremonies, most of the participants were aware of this power imbalance not just because of the small number of Palestinian participants, but also because of the Hebrew language, the particular fashion, the cultural capital, and accessibility to ayahuasca. In some ceremonies, Palestinian participants sat as one group, and were perceived – by themselves and others - as a ‘separate’ unit. Therefore, active participation of Arab-Palestinians - besides creating an opportunity for Jewish participants to connect to Palestinian culture - sometimes led to the recognition of this power imbalance, which in many cases led to inclusion as well.

Most commonly, this type of recognition was facilitated through music or prayers in which the ‘frequency’ or ‘vibration’ of each language was noticeable. Important to note, though, that this type of connection was not immediate. Many participants expressed that in initial encounters, listening to the language and songs of ‘the Other’ created resistance, fear, and anger – yet, working through these emotions was impactful.

*Arab-Palestinian woman from Israel:* “There is resistance to anything Arab, be it the language, be it a person. Although they like the Hummus. So I want to bring it through the singing, through a frequency that will touch somehow. Up ‘till now, many have told me they like my singing. They ask “will you sing to us in the night?” they like it. They feel it opens for them. Whether it’s a difficulty or a good thing, it helps them, to see. At first, there might be resistance - that releases things inside. It might cleanse, through laughter, through tears. They understand through the resistance and the journey they go through the night that it does them good.

*Jewish-Israeli woman*: “You can feel the energy is tense, but again – this is part of the work. After all, it is necessary for us to enter the uncomfortable and ‘dark’ places, and that will create resistance.”

### Connection Through Music or Prayer

As participants were able to express themselves, including their identities and background, through music, the ceremony became a space in which intercultural and interfaith exchange occurred, and this was intensified by ayahuasca. This allowed a strong recognition and appreciation of the spiritual qualities of the other culture, and it also allowed participants to feel comfortable and safe in the ceremony while listening to music from their own culture.

*Arab-Palestinian woman from Israel:* “It’s amazing. And it stays with them. That I feel in the language of my present origin, I am an Arab now, Arabic is my mother tongue. I sing from the source so it really reaches everyone. I can feel that I sing from a full connection. I feel everyone rises, I open another gate … I see there is an awakening. Something opens”

*Jewish-Israeli man:* “Arabic singing opened the hearts of Hebrew people who were in the circle, female songs of Arab women which open very powerful gates in the mind of Jewish people. At the level of readiness for peace and the enabling of peace, internal and external. A place opens in the heart to Arab culture, another aspect of its beauty”

*Jewish-Israeli woman:* “Calm. Calm. Everybody cried, everybody was very moved, by the beauty, the voice. But also the simple connection. Wow, it was very moving, the singing. The laughter … He began to sing, one of the most beautiful voices I have ever heard. In Arabic. There was silence. Ceremonies are quiet, the ones I go to. But here there was a pshhhoo, like a connection, a listening, crying. Lots of people cried. It was very moving.”

*Jewish-Israeli non-binary person:* “[The song touched me] in the place where there is fear to hear, the old pattern that says ‘oh they are singing in Arabic that’s frightening.’- that’s healing! So then you say “Wow, who did this to me, who took away my ability to enjoy that healing all these years.”

*Arab-Palestinian woman from Israel:* “In the beginning I used to criticize the idea - they used to read a lot from the Torah. So I used to put on the Quran - we also have a culture we want to share. And I remember the tears.”

### Connection Through Paraverbal Qualities of Each Language

Many interviewees describe the importance of listening to ‘the Other’s’ language and learning to enjoy it. Some described that each language has its own ‘frequency’ or ‘vibration’ and creates different visual imagery while listening to it.

*Jewish-Israeli man:* “Suddenly you hear the language you most hated, maybe the only language you really hated, and suddenly it is sending you into love and light, and that's the way it always is. Whatever the song, whatever the words, you melt- that's it that's our peace, to sit and listen to a song in Arabic, that's peace.”

*Arab-Palestinian woman from Israel:* “The frequency of the words and the letters and also where the person is singing from - it affects the listeners, who understand nothing of the letters or the words but they feel the frequency, and it’s like wow, who is singing, what is this?”

*Arab-Palestinian man from Israel* “[…] it’s more of a challenge, to enter the Hebrew language, or a language I don’t understand. And that was in the beginning before I paid attention to the vibration behind the meaning of the word. Because the meaning of the word, like every word, can be perceived positively or negatively. I learned that the heart of the word is how I receive it not how it was meant.”

### Experiencing Awe and Silence

Different interviewees described these moments of recognition as moments of ‘expansion’ or ‘opening,’ which in many occasions, comes with silent attention during and/or after. Some describe these moments like those in which the group moves from being each one in his individual process to being aware of the rest of the group. These qualities of ‘expansion,’ or silence, emphasize the importance of these moments, and the feeling of reverence and awe which they bring. In the local jargon, ‘expansion’ (*Hitrachvot, התרחבות)* also means a moment of shared or communal processes. Interviewees’ suggest that the feeling of *awe* which is associated with these moments of recognition brings hope to participants as if ‘peace was felt in the air.’

*Jewish-Israeli man:* “[..] every time a Jew would sing a religious song or an Arab would sing in Arabic, there was a big wow, but silent. Nobody would say, how lovely, a song in Arabic. Nobody would point out that something from outside had happened. There was a moment in which [the Shaman] sang a song in Hebrew or something religious in Arabic, and when he finished one of the Arabs said ’wow, you Jews know something.’ Like, you also got it, you got the groove.”

*Jewish Israeli man:* “Suddenly everything becomes silent and everyone is exhilarated. When someone sings in Arabic, I don’t know what happens in the individual processes but I do sense something powerful happening in the space.”

*Jewish-Israeli Woman:* “When someone is saying ‘Allahu Akber’ in a ceremony, one can feel how the room is flooded with love and how people burst the limits of their normal consciousness and connect to something beyond … It is a moment of great expansion – when we are in a group in a moment that takes us away from our regular frameworks, from our regular life, and transcends us to another insight, and puts in the container something that was there but that we didn’t pay attention to – and suddenly when everyone feels it and connects to more truth, to more love, then it expands the container of the group. This is a huge process of healing in my opinion”

## Conflict-Related Revelations

Many interviewees reported experiencing some form of collective trauma and grief (15/31). Importantly, although half of the interviewees reported these events, interviewees considered them rare and they occurred only once or twice within a practice which extended over many, often dozens of, ceremonies. These events usually consisted of visionary mental content, though sometimes they were through other cognitive and emotional processes such as moments of insights and catharsis. These events were sometimes related to autobiographical memories, or to more collective elements like the *land* or historical events. In most cases, interviewees reported that the presence of ‘the Other’ in the ceremony, or the location of the ceremony, are what *triggered* these mental images and processes, a notion which highlights the multilocality of these processes. Unlike Unity-Based connection which was related to ‘core-humanity’ and *universality* of identities, these revelations emphasize the history and local identities related to the conflict. In some cases, revelations inspired participants to want to share *the message* from the ‘vision’ to the rest of the group. Sometimes this was accomplished by singing to the group immediately after the ‘vision’ or during it. Interviewees reported that by doing so they attempted to reveal something and ‘bring some truth’ to the rest of the group.

### Collective and Intergenerational Revelations

Some interviewees reported experiencing collective trauma and pain related to the conflict, and some also reported intergenerational traumatic revelations. These experiences are defined as being understood as relating to past traumatic, collective, and historical events.

*Jewish-Israeli man, describing an experience which was triggered by a woman singing in Spanish about motherhood*: “Suddenly I feel for the first time that I can connect with all the fathers who lost a child in the war, in the conflict here … Suddenly I had this thought and it was also my first cry-in-public in a ceremony.” (this Israeli participant came from a strong left-wing background and said that he never before empathized with soldiers or their families as he was opposing the army).

*Arab-Palestinian man from Israel:* “[We were in (a Jewish village)], it’s an Arab village whose people were deported, and there is a [Muslim] cemetery which is probably that of those village people. And we drank and I decided to have this trip outdoors … then I began - I will never forget it - to see people rise up from the graves. Arabs. Locals … they are walking to the bus, and I start screaming to them “come back, it’s OK, no one will kill you, we are here!” and they can’t hear me. It was a very difficult moment. I see them on their way to Lebanon, just like I learned, like what I read about all my life, and I yelled to them and it didn’t help, they went … it was very, very sad. And the absurd thing is that the person comforting me is this Jewish guy (laughs).”

The same Arab man reported another ‘vision,’ which he reported as one of the most transformative events in his life, and considered this to be one of the most important experiences he had with ayahuasca. He was educated in the Jewish-Israeli educational system and considered himself to be ‘brain-washed’ up to this moment (before this he supported right-wing Israeli governments – which is unusual for an Arab-Palestinian). In both of his experiences, the visionary mental content was *triggered* by and related to the location of the ceremony.

An interesting moment was the first ceremony in [a Jewish city in Northern Israel] in an Arab house with a [Jewish] religious family living in it (in Israel many homes once belonged to Arab families who were forced to flee during the 1948 war. Israelis, most often Jews, live in them these days)… I went outside [and sat under a vine tree which reminded me of my grandfather’s house] and suddenly I had a very strong vision. I see - there was a balcony- and these are houses like my grandfather’s in the village, old Arab houses. And there it hit me. It hit me and I began to see, I saw the [former] owners - an old man like my grandfather and an old woman like my grandmother with a scarf on her head, I started to interview them, we started a dialogue. And that talk, today I describe it as it washed out all the brainwashing I had gone through as a teenager, all of the Israeli school, and the youth movements … I connected to being Arab, and it was a serious shock for me … [They were] old, with traditional Arab clothes, head scarfs and all that. They were good people, they caressed with their words. The woman mainly spoke. She said “I know you went to an Israeli school, that you hate everything to do with Arabs and Arab things, and you have anger and this and that. But the story is different. You heard one side but you haven’t heard our side. You are in our house now, and we are not here. We were evicted … and that’s when all the things began to evaporate. But it didn’t take me to the other side, didn’t make me hate the other side.”

When the above ‘vision’ ended, he returned to the group, angry at himself, and started singing in Arabic in order to disrupt the ceremony. The shaman then invited him to sit next to him, and started to sing Lebanese songs of Fairuz and other folklore. At that moment “everybody gets up and is dancing and hugging.” The multilocality of this event is highly diverse: it took place in an old Arab-Palestinian house which is now owned by Jewish people in a religious town, and the music was Hebrew. There was a vine tree that reminded him of his grandfather, and this ‘vision’ of historical trauma was related to his collective identity. After his ‘vision’ ended, he intervened in the ceremony process, – an intervention which emphasized collective identities of all participants and which eventually enhanced the intercultural connection and the recognition of historical injustice.

### Seeing the Other Side

Some interviewees described moments of strong empathy with the trauma of ‘the Other,’ and sometimes this was in the form of a visionary mental content.

*Arab-Palestinian man from West-Bank:* “I had this weird experience of being in the body of an Israeli soldier. It was like seconds of experience – the whole experience was the eye coming down to look for shooting and as the trigger is pulled, that’s it, there is no seeing after…..I could feel him after, this is painful, this is not an easy life after.”

*Arab-Palestinian woman from Israel, describing a vision of a past life as a Jew*: “But in this past life, my brothers and sisters were Jewish, and I died in the war, and I saw myself in this Cycle, in 1918 I was a Jewish fighter, I saw myself and they killed me.”

*Jewish-Israeli man, who was in an elite combat unit in the past, describes a vision in which he sees himself doing a house arrest and then re-experience everything from the side of the Palestinian family. The vision is triggered by a group of Palestinians which cry in the ceremony*: “I heard him cry, they were all sitting together, suddenly the Ayahuasca showed me them as a separate unit within us and they began, another one began to cry, and it took me automatically to the madness of the pain of a whole people, I could see it, it was clear to me that they weren’t like weeping because they remembered a dead aunt or something, they are weeping the pain of their people, and I am connected to that pain. I caused the pain of their people, I began to break, I couldn’t stand to hear them crying and she (the Ayahuasca) began showing me so much, I can't describe it visually, just this crazy pain and hate and crying for the evil they experienced, and it built up and up till there was a cut. It was one of the most powerful experiences I had, in which Ayahuasca took me and put me in a situation that seemed quite real to me and separate from the room I was sitting in - I get Goosebumps just thinking about it. I just saw myself in a jeep on the way to an arrest ... I see me entering with my officer, and we close on the house and start making announcements to get the person out of the house, and what happens is that the door opens, we see an entire family sitting in the living room and screaming and banging and someone trying to run, and you just go into the house and catch everyone there, sit them down, try to find him out … In total hysteria and panic we manage to get him into the car, get out, the arrest is over and we turn him over to some GSS investigator. And this is exactly what the Ayahuasca is showing me, and as it is over, and when we get out of the car with him there is a cut. And now I'm just sitting with them in the living room. It like went back in time, I am sitting with the family in the living room and I see them drink coffee, talking and laughing, the grandfather plays with his grandson, they're happy, the TV is in the corner. And everything is good, then suddenly there is pounding at the door, it was very weird, I don’t understand what's happening, I see the guy I'm supposed to arrest running from the room, then the grandmother or someone opens the door, and I see me, like Robocop or something, with a ski mask, I can see my eyes clearly, I look at me and I really freeze in fear, I feel the heartbreak in this second in this room, all that happened in this room in this instant, and the crazy fear people are feeling. Now I am looking at me, and I can't believe I am the person standing there, I look horrible, In my eyes I look like something from a film about Nazis … I really felt the hate towards me build. [I saw myself arresting the guy], leaving, and the door closes, and I stay with them, a broken family whose world has just been destroyed, and I just see the little brothers and it's clear we just raised three generations of terrorists who will definitely hate me, and my eyes in that ski mask might be carved for life in their minds as the eyes of the guy who ruined their life, and it undid me, completely, like I'm almost crying now, it was the first time I felt - I hated myself, deeply I mean, I was angry at myself and hated myself and said, it makes no difference, Israel Palestine, whatever, I can't believe I did that.”

When he ‘returned’ from his ‘vision’ back to the ceremony, he reported a moment in which he ‘channeled’ a Hebrew ‘icaro’ (song) to the rest of the group, which addressed the ‘sacred’ connection to the land. The interviewee was aware that the word ‘channeling’ is sometimes used inauthentically, but stated that in his experience, he actually felt that way. He reported that it felt like he had no choice but to sing, and that this moment felt uncontrollable. He considers the song part of a continuous process from the ‘vision.’ He sang with full confidence, as a “preacher-man”, and felt that “truth came out of [him]” when he sang. The words of the song emphasize recognition and connection to the land (from Hebrew): “My place on the land is sacred; water and sky – here my voice is heard; and around me the great spirit”. The song reduced his self-hatred, which had been induced by the ‘vision,’ and released tension in the group. This was the first time he sang a song in a ceremony. He considered this event to be a strong, personal, transformative moment in relation to the conflict; for example, afterward, he started to study Arabic. Others who participated in this ceremony also describe this ceremony as very transformative in healing the relations between Jewish Israelis and Palestinians who were together in the ceremony.

A Palestinian who attended this event, and was part of the Palestinian group crying together, reported that it was not a Palestinian who started their collective cry, but their other Israeli friend who sat next to them and was processing his own traumatic experience from the army, and they cried with him (ambivalently), as they knew his history in the army. Again, the multilocality of this story is evident – an Israeli cries about his army-related trauma, this moved his Palestinian friends to cry as well, this collective cry triggered a ‘vision’ in another Israeli, the ‘vision’ is experienced from both sides in the conflict, after the ‘vision’ ends, he sang a song to the group which channeled ‘emancipatory truth’ and released the tension.

### Seeing the Pain of the Land

Multiple Palestinian women described intense visionary revelations related to the pain of the land, in which there is a description of bloodshed into the land.

*Arab-Palestinian woman from Israel, in a women-only ceremony:* “I could feel what the earth feels, what it hurts, what it needs. There was a moment where all the women sang together (this occurred in a women circle), sacred songs that I did not know the words to, but the tone and the singing and the female voice, and like this I simply got connected to the earth and I saw it absorbed with blood under us, and I realized that she was suffering and she was asking for healing, and I saw the female-singing drying the blood out of the ground, and I started to act as if I’m leading and I asked to “continue and continue! enormous healing is taking place, which I see and feel”. In some moment they stopped singing and I told them “please continue, continue” and they all burst in laughter – not understanding what is up with me.”

The next example demonstrates again how conflict-related revelations can sometimes inspire the need to deliver *the message* to the rest of the group.

*Arab-Palestinian woman from Israel:* “It was on *Yom Kippur* (Day of Atonement, the holiest day in Judaism), and out of me came the opening paragraph of the Quran … and another frequency opened up, and I understood how much pain is in Pachamamma, mother Earth, how it hurts her all the red in this country, the blood. How much pain, how much anger. When I sang it I was fully present, no right or left, it came from my centre, in a voice of - I can’t say in the voice of God - but in the voice of a messenger, listen and awaken, there is a battle here between light and dark. The dark is growing more than the light, so understand where we are now. Even the shaman that night said to me “wow, you went so high”, and from there something opened. In that session they were vomiting and when I was done, they said we have to talk to you in the morning and they said how their understanding grew, we are not here just to … I felt each one, each and every one, where he was, with his fears and his doubt, and his ego, where he is, the net each person places himself in. The defense of the illusion. There are defenses, yes. We arrived protected, what are you afraid of. I will not forget this session. There was something of - understand, understand. And it came through me... it was a very strong vision, very painful, and after that I couldn’t stop crying”

## Discussion

In this study, we observed three types of *multilocal participatory events* (Ferrer, 2002) that occur during ayahuasca ceremonies with mixed groups of Israelis and Palestinians. These events consisted of elements that were intrasubjective, intersubjective, communal, related to collective identities, and related to places and the land. The two most commonly reported events represent types of connection that are distinct from and yet complete each other. The first was a felt universal connection among group members, in which *oneness* and *unity* are felt by the group and individuals within it. The second observed connection was based on diversity, in which participants related to each other based on non-*universal* collective identities (Muslim, Christian, Jewish, Arab, Israeli, Palestinian). This connection usually occurred through music or prayer, which supported an intercultural exchange, and these moments were marked by the silence which followed them and a feeling of openness, reverence and expansion. The third theme - conflict-related revelations - was rarer. However, when it did happen, it was considered as a very unique and transformative event, to both individuals that had these revelations and to the group when the participant attempted to ‘deliver the message’ of the revelation to the rest of the group. All of these themes have participatory and relational elements which will be elaborated throughout the discussion.

The first observed theme was connection based on similarities, and out of this theme, the most evident connection was a felt universal connection *beyond identities* which was related to moments of *oneness* and *unity.* The description of these moments of *oneness* is similar to the Victor and Edith Turner description of communitas, which is a group event in which social structures and hierarchies collapse, and people experience each other based on shared humanity (Turner, 1969/2017; Turner, 2012). These were crucial moments for the group, and the feeling of *unity* among group members was relevant not just to the acute state, but also to the formation of ‘tribal’ relations between group members that emerged. *Communitas* has already been observed in a large online study, and was found to moderate long term changes in well-being and social connectedness (Kettner et al., 2021). These events of group ‘oneness’ and identity-dissolution can be perceived as the social manifestation of other well known psychedelic experiences such as the mystical-union and ego-dissolution (Pahnke and Richards, 1966; Shanon, 2002; Loizaga-Velder and Verres, 2014). Overall, interviewees highly valued these moments of ‘oneness,’ and they consider them to be of significance in healing intercultural and interfaith relations, and in peacebuilding.

However, it is suggested that communitas sometimes can paradoxically reinforce social structures by providing a momentary cathartic release from them (Turner, 1969/2017). A similar criticism is directed at reconciliation-aimed intergroup encounters that over-emphasize similarities between groups embedded in asymmetrical conflict (Maoz, 2011). Commonality-focused workshops that ‘equalize’ everyone may in fact serve to *reinforce* rather than challenge the inequality which takes place outside of these workshops (Yanay and Lifshitz-Oron, 2003; Saguy et al., 2009; Schimmel, 2009; Zreik, 2016). Only a well-rounded methodology which focuses on both coexistence and confrontation – unity and diversity - can address systemic concerns along with personal connection (Abu-Nimer et al., 2007). Therefore, the second and third themes (recognition, and conflict-related revelations, respectively) are of importance if psychedelics are used for peacebuilding, as they relate to intercultural and power differences (second theme), or directly related to the conflict and its history (third theme).

The second theme to some extent contrasts with the first theme. Interviewees reported recognition and connection based on difference, inspired by an intercultural exchange based on local identities. This usually occurred through music or prayers, and is described as a unique moment where what is not expressed verbally is expressed musically. Music is considered ‘the hidden therapist’ in psychedelic therapy (Kaelen et al., 2017) and is crucial in ayahuasca traditions (Katz and De Rios, 1971; Bustos, 2008; Luna, 2011; Molina, 2014) and in exerting cultural influence on the visionary mental content (Langdon, 1979). Furthermore, singing can facilitate participatory spirituality (Freinkel, 2015), and this was crucial for cultural exchange to take place in the observed ceremonies. Interviewees mentioned that they resonated with the ‘vibration’ of each language music. ‘Vibrations’ between participants have been observed among ayahuasca neoshamanic groups elsewhere (Gearin, 2016a), and in this study these ‘vibrations’ were related to intercultural exchange and recognition as well.

Moments in which participants resonated with the music of ‘the Other’ culture were described as spiritual moments of openheartedness when ‘peace’ was felt ‘in the air.’ Furthermore, moments when Arab-Palestinian participants sang were marked by the group with an attentive silence, and some described a feeling of expansion in which individuals ‘woke-up’ from their own individual process and become more attentive to the group process. The reported collective uniqueness of such moments implies that communitas existed in these moments as well, and some interviewees even used the word ‘oneness’ to describe these moments. This momentary group ‘peak’ process might be related to a sense of *awe* which is associated with the act of recognition and the inclusion of what was previously excluded from mental structures (Keltner and Haidt, 2003), in a similar way to “aha” moments of insight (Kounios and Beeman, 2009). A lack of recognition can manifest as a form of oppression (Taylor, 1997), and the *universalistic* ideology which the ceremonies are framed around can exclude the importance of the Palestinian history and struggle. As *universalism* can be - in many cases - the hegemony of the particular (Taylor, 1997; Saldanha, 2007; Wolosky, 2016), it can lead to the exclusion of minorities, such as in the case of the ‘identity-blindness’ of Israeli new-age (Simchai and Keshet, 2016). Therefore, when this ‘political’ aspect ‘leaks’ in the ceremony, there is a moment of inclusion of something that was concealed by new-age ideology.

From a theoretical perspective, power-inversion is known to be one of the primary ritualistic methods to reach communitas (Turner, 1969/2017). Therefore, the singing of the Arab-Palestinian minority, can lead to a perceived role-inversion and *anti-structure*, and therefore to a state of liminality and its associated communitas. As mentioned before, communitas can sometimes serve as a cathartic moment from the social structure without necessarily changing it, and even strengthening it. Therefore, it is important to note, that many of the Arab-Palestinians participants who received recognition through singing, later on became facilitators or helpers and are well-respected within the observed groups. That is to say, that these moments of recognition were not only localized to the ceremony, but also transformed the role of Arab-Palestinian participants within the groups.

Ethnomusicology has been used in order to study the diffusion of ayahuasca within the Amazon (Brabec de Mori, 2011). Since the rubber boom, ayahuasca practices swiftly diffused and diversified between different ethnic groups and cultures (Gow, 1994). The spread of ayahuasca within the Amazon and across the globe is also the spread of music. When ayahuasca practices move from one group to another, musical forms move as well, and each new ayahuasca practice incorporates its own musical preferences onto the musical heritage with which ayahuasca was received. Therefore, music is at the center of this intercultural and interethnic exchange.

In the case of the current investigation, Arab-Palestinian participants were mostly newcomers into a Jewish-Israeli ayahuasca practice which was already established for a number of decades, with its own Jewish-Israeli musical influences. Moments of Arabic singing or prayers within the ceremony were also moments of an intercultural novelty for many who attended such ceremonies. The awe and reverence which characterized some of these moments of recognition, might be a crucial element in the international, intercultural and interethnic diffusion of ayahuasca. This form of relation to ‘the other’ can be considered as exoticizing ‘the other’ (Taussig, 1987; Fotiou, 2020), but nonetheless, this alterity can also support the healing of oneself and one’s own culture, and therefore might be crucial for considering ayahuasca as an intercultural bridge (Langdon, 2013). While *initial* encounters with ayahuasca are encounters with alterity, the need to recognize one’s own background within a continuing practice can eventually lead to modifications of the practice to accommodate participants, and diversify the practice to support diffusion. Singing in a ceremony is also a way in which one practices his own ‘shamanic powers,’ and it is an initial step in the path of becoming a facilitator. When Arab-Palestinian participants join ceremonies in Israel, they are dependent on Jewish-Israelis to support and facilitate their process. The observed moments of recognition through singing are also moments of independence as the one who is ‘being healed’ is now becoming a ‘healer.’ Within the Amazon, when the colonized becomes a ‘healer’ a new form of relationship is established which can transcend – to some extent, but not completely - the old extractive and abusive mentality of the colonizer-colonized relation (Saéz, 2014; Fotiou, 2020) - and this notion which might be true to the Amazon is glimpsed in the middle east.

This second theme fits the spiritual framework of Martin Buber, which describes the spirituality of ‘the between’ in his seminal book ‘I and Thou’ (Buber, 1923/1970). *I-Thou* relationships are relations for the sake of relation, without objectifying, and without ‘using’ each other. Buber emphasized the importance of duality and communion of these relationships, and that what is needed to enter these types of relations is not a dissolution of the self, but a dissolution of the need for self-affirmation. In *I-Thou* relationships, differences are vital for growth through dialogue. The interviewees’ descriptions of these states resemble *I-Thou* relations both in their spiritual undertone, and because these relations were based on differences of identities and not just similarities. These difference-based relations are known to be a vital component for interfaith and dialogue encounters (Abu-Nimer, 2001; Kuttner, 2012), and these relations were intensified by ayahuasca. In many cases, interviewees suggested that in initial encounters, differences in identities might have led to feelings of fear, anger, shame, guilt or embarrassment, and that the transformation of these emotions was not immediate. Moments of *I-Thou* intercultural relations served as moments of transformation or ‘breakthrough,’ which appeared to be vital mediators of longer-term shifts in perspective about the other culture outside of the context of the ceremony. Similarly, Ron and Maoz (2013a) describe how encountering the stories and experiences of 'the Other' in the context of an intergroup dialogue can be a transformative form of intergroup engagement as it creates spaces for different affective and ethical relations with others. Furthermore, it is important to mention that Buber emphasized that *I-Thou* relationships can take a spiritual form, which is distinct from the descriptions of the *mystical-union,* by virtue of maintaining duality. *I-Thou,* therefore, may be another characteristic of the psychedelic state that can play an important role especially in relational healing.

The third theme was conflict-related revelations associated with collective pain and trauma, the pain of the ‘other’ side, or the pain of ‘the land.’ Biographical and historical traumatic ‘visions’ are known to be part of the phenomenological repertoire of ayahuasca (Shanon, 2002; Kjellgren et al., 2009; Trichter et al., 2009; Fernández and Fábregas, 2014; Loizaga-Velder and Verres, 2014; Apud, 2020a) and other psychedelics (Frederking, 1955; Belser et al., 2017), and are considered therapeutic in psychedelic-assisted therapy, e.g., Jan Bastiaans’s LSD therapy for Holocaust survivors in which ‘visions’ from the Holocaust were considered part of the therapeutic modality (Snelders, 1998; Enning, 2009). Yet, what was key in our investigation was not just the content of the reported ‘visions,’ but also how the immediate environment and locality impacted these ‘visions,’ and how these ‘visions’ affected the environment in return. In many cases, the revelations were *triggered* by the presence of ‘the Other’ in the group, by music, or by the location (e.g., a house that belonged to Palestinians who were forced to flee), or time (*Yom-Kippur)* of the ceremony. In some situations, the revelations led the participants to attempt to ‘deliver’ the insight to the rest of the group. What is evident in these revelations is that they are multilocal – it is a process that includes the individual, the group, identities, intercultural and interfaith relations, the place and time, and the land of Israel/Palestine. These revelations can be *triggered* by an element interpreted to be related to the conflict, and they can lead the participant to address the group with an emancipatory message related to the conflict. Collective identities are emphasized, and they are not merely universal – they include Palestinian, Israeli, Jewish, Muslim and Christian history, and participants experience these revelations from the lens of these identities. This finding of traumatic revelations is aligned with the well-known potential of psychedelics and empathogens in treating PTSD (Nielson and Megler, 2014; Mithoefer et al., 2018; Krediet et al., 2020). However, these treatments, and treatments of other conditions, are focused on the *inner* processes (e.g., ‘inner-healer’) in which it is suggested that the patient can treat himself by ‘letting-go’ and focusing on his own psychedelic process *within*. In the traumatic revelations observed in this study, a relational process occurred *between* participants as well. Visionary mental processes were *triggered* by the presence or music of other participants, and participants sometimes responded to these ‘visions’ by addressing the group. Therefore, the therapeutic process did not occur only within participants but also between them, and within the group. As trauma is in most cases a relational wound, addressing it relationally might be more beneficial to both individuals and society at large (Bistoen et al., 2014). Healing trauma collectively can also be more effective in building trust for the long term.

The participatory and relational elements which were observed in this study challenge the consensus within new-age (Simchai and Keshet, 2016)*,* transpersonal psychology (Ferrer, 2002) and the psychedelic movement (Baier, 2017), that spirituality consists of mainly *inner* experiences. (Though, it is important to acknowledge that there are diverging streams within new-age (Heelas, 2009) and contemporary ayahuasca culture (Gearin, 2016a) which do focus more on relational and reciprocal elements of spirituality.) This inner-spirituality framework was also held by most of the interviewees through association with new-age, though the participatory nature of the ceremonies allowed the emergence of events which did not necessarily fit their universal ideology. A clearer understanding of such relational elements alongside personal elements can serve to support the integration process after the ceremony or therapy by (ideally) thwarting spiritual bypassing (Masters, 2010) and spiritual narcissism (Lasch, 1979/2018), and by reducing intrasubjective reductionism, which can inspire anxiety and alienation through an extensive focus on one’s ‘self’ without recognizing social factors of mental health (Rothberg, 1993; Ferrer, 2002). The psychedelic state is in many cases an enhanced version of the ‘real-life’ state (Grof, 1975/2016), and creating a framework in which these relational elements can be discussed can clarify the same elements after the psychedelic induced sensitivity is reduced.

The overemphasis on *inner* personal processes is natural when psychedelics are considered therapeutic agents – the intention of western participants in ayahuasca ceremonies is psycho-spiritual growth, and therefore ayahuasca facilitators perceive themselves mostly as alternative therapists, and the financial exchange is with this therapeutic intention in mind. However, while psychedelic therapists, neoshamans and facilitators are encouraging participants to ‘go inward,’ they are intuitively well aware of other relational processes. Increased suggestibility (Carhart-Harris et al., 2014), the effect of the music on the therapeutic process (Kaelen et al., 2017), and the rare intimacy that is developed with the therapists (Watts et al., 2017) are all relational processes. Focusing on an *inner* language to describe the therapeutic process creates a façade of individual independency, while it is reasonable to assume that some of the personal-growth that is achieved in these processes is achieved by intimacy, connection and interdependency. Furthermore, through having psychedelic experiences, one is *initiated* into a wider milieu, and/or local communities, which can help fulfill humans’ need for belonging, and therefore may address a relational cause of mental health (Hari, 2019).

If these relational processes are not acknowledged, integrating them in real life is harder to achieve; e.g., if one does not see the importance of the communal environment of ayahuasca ceremonies, one will not try to fulfill this need for a community outside of the ceremony. Building resilience on the personal level, or finding ‘spiritual connection,’ does not automatically mean supporting the creation of a better life-context in which some of the social causes of mental health concerns are reduced. However, we believe that by nourishing a relational-participatory framework, the therapeutic process can impact not only individuals, but society as a whole, and the potential of psychedelics *beyond therapy* will become clearer.

Before concluding, a few limitations of this study must be acknowledged. The study has focused on specific neoshamanic practices which incorporate participatory elements. One cannot generalize from this study that any ayahuasca practice can support peacebuilding processes. For example, the second theme of recognition and difference-based connection, requires active participation in which participants can ‘share’ songs or prayers of their choice. Many ayahuasca practices do not have this element, and therefore it cannot be assumed that this relational process would occur in such practices. Another limitation was that while the study aimed to investigate the potential for peacebuilding, the investigated groups were not formed around this intention. The motivation for participation in these groups was for psychospiritual reasons. Personal mental-health of participants was listed as a top priority for avoiding introducing more politics into the ceremonies, as it was considered a risk which could sacrifice the harmony and mental safety in the ceremony. Future investigations should examine groups who intentionally meet for peacebuilding and dialogue, and groups with a more even balance of Palestinians and Jewish Israelis. Another limitation was a self-selecting bias of the population of the study. Those who were willing to be interviewed for a peacebuilding study might be those with a certain sociopolitical and spiritual understandings. Furthermore, ayahuasca is an illegal practice in many of the studied groups and this has limited our study population only to participants who were willing to ‘take the risk’ and trust us. The research population was relatively homogenous, and came from a social upper middle class, and were part of the new-age milieu. It cannot be concluded that the observed process could be extrapolated to a wider population.

When psychedelics are used for therapy or ‘psychospiritual growth,’ the interpretive framework focuses mostly on personal concerns; therefore, approaching psychedelics as tools for peacebuilding requires acknowledging the potential limitations of inner-spirituality for social change. Everyday inequality and sociopolitical tensions must be factored into both peacebuilding frameworks and individual therapeutic frameworks alike. As momentary harmony between those in conflict can sometimes serve the status-quo (Saguy et al., 2009; Hasan-Aslih et al., 2019), peacebuilding should not be seen as only achieving a state of harmony, as many interfaith and dialogue groups try to achieve, but also engaging in political liberation of those who suffer from political injustice (Abu-Nimer et al., 2007).

The observation in this research reveals that while psychedelics can create group harmony and communitas, they can also lead to experiences and events directly associated with sociopolitical awareness. Such revelations and hallucinatory states – regardless of psychedelic use – have a long history in igniting movements and shifting sociopolitical structures (Mooney, 1896/1991; Wallace, 1956; Taves, 2016). As observed in this study, though personal and group experiences might have sociopolitical undertones, if they are still framed by participants through an ‘apolitical’ therapeutic framework, participants often dismiss or avoid examining their sociopolitical importance. Therefore, intentional and impactful psychedelic-assisted peacebuilding should be grounded in a framework which includes political liberation; not just by ‘freeing one’s mind’ (Karkabi, 2021), but also by attempting to change material reality. We theorize this shift will invite more integration of historical or political revelatory *events*, and moments of recognition, into the larger sociopolitical reality (Badiou, 1988/2007; Roseman and Karkabi, 2021).

## Data Availability

The datasets presented in this article are not readily available because of sensitive information which includes illegal use of psychedelics. Requests to access the datasets should be directed to leor.roseman13@imperial.ac.uk.

## Footnotes

1 Many names exist for the person who facilitates ayahuasca rituals and healing e.g. curandero, onaya, maestro, ayahuasquero, vegetalista, taita, etc. In this manuscript, we chose to use the word shaman, as this was the name used by most of our interviewees who were participants in what could be called a ‘neoshamanic’ ceremony (Scuro and Rodd, 2015). We acknowledge, however, that ‘shaman’ is a name given by westerners to Amazonian practitioners and it actually originates from Siberia. Yet, important to note, that although the term shaman might be problematic, it is also the name currently used by many indigenous and mestizo people, and shamanism can be seen as a dynamic practice that was developed, and is still developing, through the interaction between western, indigenous and mestizo/caboclo people (Fotiou, 2020).
