# Supplementary material for: Relational Processes in Ayahuasca Groups of Palestinians and Israelis
Source: Front Pharmacol. 2021 May 19;12:607529. doi: 10.3389/fphar.2021.607529 (PMC8170481; doi:10.3389/fphar.2021.607529)
Supplement: Supplementary file 1 [file datasheet1.docx]

# Supplementary Material:

# Altered-States of Contact: Relational Processes in Ayahuasca Groups of Palestinians and Israelis

Leor Roseman^1^, Yiftah Ron^2^, Antwan Saca, Natalie Ginsberg^3^, Lisa Luan^1^, Nadeem Karkabi^4^, Rick Doblin^3^ Robin Carhart-Harris^1^

^1^ Centre for Psychedelic Research, Imperial College London, London United Kingdom

^2^ Faculty of Social Sciences, Hebrew University, Jerusalem, Israel

^3^ Multidisciplinary Association for Psychedelic Studies (MAPS), Santa Cruz, USA

^4^ Anthropology Department, University of Haifa, Haifa, Israel

Correspondence:

Leor Roseman

Leor.roseman13@imperial.ac.uk

## Initial Inquiry

An initial unrecorded focused interview was conducted of a mixed group of 15 people from one ayahuasca group. This focus group interview (Morgan, 1996) was conducted a year before the rest of the official and recorded personal interviews, and it served as an initial inquiry into the field of research which supported the formation of the semi-structured interview. The focus group was interviewed by LR and NLG. Furthermore, initial unrecorded conversations took place with seven different participants from different groups by LR, for the same purpose.

## Semi-structured interview

The in-depth semi-structured interview took place face to face and was between 1 hour to 2.5 hours. We had an initial connection to a few interviewees, and after trust was attained during the interview, they have linked us to the next interviewee in a rolling manner. We stopped interviewing after achieving saturation (no new information being gathered in an interview). The sample was a *purposeful* sample in which we attempted to choose diverse informants in order to provide us with reliable and trust-worthy information (Mason, 1996). Interviews discussed a range of past experiences which sometime was over few years of practice. Most of the interviews took place within a month period in which LR and AIS were traveling across Israel/Palestine and in which NLG joined for a few days. LR conducted 20 interviews (15 Jewish - 14 in Hebrew and 1 in English; 5 Palestinians - 4 in Hebrew and 1 in English), AIS conducted seven interviews (all Palestinians – 5 in Arabic and 2 in English), and NLG conducted four interviews (3 Jewish, 1 Palestinians – all in English). Qualitative analysis of interview data tends to begin during the process of data collection (Charmaz, 2000), and in the case of this study this was done in a dialogical way between LR, AIS and NLG in the period of data collection– that was useful as different interviewers have different backgrounds which likely affected their interpretations (Shkedi, 2003). This dialogical process enables a more complete picture of the field of inquiry – LR is Jewish-Israeli with background in psychedelic research, neuroscience and mental health, AIS is Christian-Palestinian, from the West Bank, with a background in political activism and reconciliation groups, and NLG is American-Jewish with background in Psychedelic policy and advocacy, mental health and social work.

The interviews themselves were split into 4 parts: 1) background, 2) questions about general ayahuasca use, 3) questions about ayahuasca in an Israeli-Palestinian context, and 4) a dialogue – using a possible bank of questions – in which the interviewer was able to express his/her thoughts, and also to challenge the interviewee in a number of topics by using stimulating and contrasting questions, by discussing contradictions in their own narrative, or discussing contradictions between their narrative with narratives of other interviewees (Shkedi, 2003). After four interviews, some questions were adapted or removed. All Arabic interviews and nine Hebrew interviews were translated into English to allow different authors to read the transcripts. Follow-up interviews and informal conversations took place for 14 interviewees, up to one year after the interview, in which some new information was received, and in which we inquired whether the interview itself served as a type of intervention that changed their future experiences.

## Coding

Analysis of data was based on (Shkedi, 2003, 2019), which is influenced by Grounded Theory (Charmaz, 1983) – which is a hypothesis-free bottom-up method of analyzing qualitative data - yet for a more descriptive purpose and not necessarily to build a theory upon the analysis. Narralizer was used to organize and code the interviews (Shkedi & Shkedi, 2005). Narralizer is a simple software for organizing and structuring qualitative data, and no automatic analysis was conducted. Each interview was read twice by one assessor, to identify initial themes. Some themes emerged independently of this immersive reading via conversations between LR, AIS, and NLG during the interview process. Complete first-level *open coding* (Strauss & Corbin, 1990) from eight transcripts was conducted, in which interviews were chosen based on the richness of content, articulacy of interviewees, and diversity between interviewees. Second-level focused coding was conducted on all interviewees after the main themes have been identified. Second-level coding was repeated by LL on a subsample of 18 interviews. LL was not part of the data gathering and her coding served to validate the chosen themes.

## Ayahuasca circles

In Israel, the facilitator distinguishes the style of the ceremony as either ‘Brazilian’ or ‘Peruvian’. The former – which is influenced by Brazilian ayahuasca churches - are ceremonies done in light with strong communal emphasis and communal singing. The latter – which is said to be influenced by Peruvian Amazonian traditions – are ceremonies done in darkness with a strong emphasis on inner, personal experiences, and in which participants usually lie down with eyes closed. In reality, these styles are not always kept entirely distinct, and in many ceremonies, elements from both styles feature. Most of the groups we investigated belong to a flexible ‘Peruvian’ style, in which there is a focus on *inner* and personal experiences. Furthermore, besides listening to facilitators or singing together, in most ceremonies there is an opportunity for other participants to express themselves through singing or other means, and this usually occurs after the peak effects of ayahuasca have subsided. This phenomenon appears to be important for many people, who suggest that ayahuasca has ‘thought’ them how to sing. Most of the participants describe the first time they sang in a ceremony as being a pivotal moment. Furthermore, this is one of the main ways in which participants achieve recognition from other group members and become ‘initiated’ as part of the ‘tribe’. Music plays a crucial part in people’s processes and is typically eclectic, including Amazonian and Mesoamerican ‘icaros’ (i.e. ceremonial chants or songs) as well as songs inspired by ayahuasca, San Pedro and Peyote ceremonial traditions; ayahuasca songs in Spanish and Portuguese; Muslim, Jewish, Christian, Buddhist and Hindu prayers; Songs from Rainbow Gatherings; and Western songs from popular culture (e.g. Stevie Wonder, The Beatles). Participants are encouraged to follow a certain diet and period of abstinence before joining a ceremony. This usually begins between 2 days to 1 week prior to the ceremony. It typically comprises of a simple, vegan diet, no alcohol or other psychoactive drugs, and no sexual intercourse. Integration circles sometimes take place on the day after the ceremony in which participants can share from their experiences, events and insights. Participants pay to take part in ceremonies and prices per ceremony vary from around $50 to $200.

## Facilitators and Groups

Our interviewees derived from a number of ayahuasca groups. Each group is led by a different facilitator or Shaman (in Hebrew the word מגיש (Me’gish) is used, which literally means ‘server’ – as the one who serves the tea). Many of the interviewees have drunk with a few of these facilitators but associate themselves with one particular group (‘tribe’). The facilitators of the different groups are (1) A Jewish-Israeli woman trained in the Peruvian ayahuasca traditions (2) A Jewish-Israeli man, trained as a Shaman in the Peruvian Amazon. A mixture of religions inspire his ceremonies and although many of his songs are from Jewish and Israeli tradition, other people from the *tribe* can sing songs from their own tradition. Many participants from this *tribe* who have been drinking for a few years became facilitators of their own groups. (3) An Arab-Palestinian man from Israel who has a wide background in different spiritual practices. (4) A European man with background in Buddhism. (5) A ‘Universal’ man (A person from a Jewish background who currently defines himself as a universal human being) with ties to rainbow gatherings, and local ‘sacred singing’ (a form of gatherings which occur in different places in Israel in which a large group of people sing spiritual songs together). In most groups there was some participatory nature and there were parts of the ceremony in which other participants can share their own music or prayers. In most of the groups, there was a minority of Arabs (around 20% of the participants or less), and very few of the ceremonies had equal representation. The Jewish participants were usually more experienced with ayahuasca as they have more access to it, and because new-age culture is more popular within Jewish-Israelis (Simchai & Keshet, 2016). This is another factor in the asymmetry of the groups as Jewish-Israeli facilitators and participants had a larger (sub)culture capital (Bourdieu, 1979/1984; Thornton, 1996). Gender representation was balanced. All of the ceremonies were conducted with the intention of personal-spiritual growth, and none of them were conducted with reconciliation or peacebuilding as the primary intention. Therefore, political topics were rarely discussed among group members. Most of the ceremonies took place in Israel, close to nature, while some took place in Palestine, Europe, Peru and Brazil. Many of the interviewees mentioned that while there is an attempt in Israel to keep the ceremony space ‘apolitical’, political reality tends to leak into the space by different forms – by the noise of a fighter aircraft, by prayers from an Arab mosque nearby, by being conducted in a Jewish home which used to be Palestinian before ‘48, or by memories of military operations or training e.g. when the ceremony is in a space in nature that is/was used for military operations.

## Interviewees’ background

Thirty-one participants were interviewed, out of which 13 Arab Palestinian (5 women, and 8 men; 7 from Christian background, and 6 from Muslim background; 9 have Israeli citizenship and 4 live under occupation in the West Bank) and 18 Jewish Israeli (8 women, 9 men, and 1 non-binary). Like many psychedelic users, interviewees came from a middle to high socio-economic background. Most of the interviewees had some experience with psychedelics or other spiritual practices before they encountered ayahuasca. In general, most of the interviewees were relatively experienced with ayahuasca and many of them served as ‘helpers’ or musicians in ceremonies. Twelve of the interviewees were facilitators or therapists themselves, using ayahuasca or psilocybin mushrooms. While the groups were diverse, they were not formed with conflict resolution as their primary aim. Therefore, our interviewees are from all walks of life and different political backgrounds, though could be described as ‘left-leaning’. Many of the Arab-Palestinian interviewees mentioned that they did not purposely choose to drink with Jewish-Israeli people, but that was their only option as there are fewer Arab facilitators (something that has changed in the last few years), and if they had an option at the beginning to drink in a group of purely Arabs, they would have chosen to do so.

## References

Bourdieu, P. (1979/1984). Distinction. Translated by Richard Nice. *Cambridge, MA: Harvard Univer*.

Charmaz, K. (1983). The grounded theory method: An explication and interpretation. *Contemporary field research*, 109-126.

Charmaz, K. (2000). Grounded theory: Objectivist and constructivist methods. *Handbook of qualitative research, 2*, 509-535.

Mason, J. (1996). Qualitative researching Sage: London.

Morgan, D. L. (1996). Focus groups. *Annual review of sociology, 22*(1), 129-152.

Shkedi, A. (2003). Words of meaning: Qualitative research-theory and practice. *Tel-Aviv: Tel-Aviv university Ramot.(Hebrew)*.

Shkedi, A. (2019). Introduction to Data Analysis in Qualitative Research: Singapore: Springer International Publishing.

Shkedi, A., & Shkedi, Y. (2005). Narralizer: A software for qualitative research analysis. *Yakum, Israel: Yazamut Yakum*.

Simchai, D., & Keshet, Y. (2016). New Age in Israel: Formative ethos, identity blindness, and implications for healthcare. *Health:, 20*(6), 635-652.

Strauss, A., & Corbin, J. (1990). *Basics of qualitative research*: Sage publications.

Thornton, S. (1996). *Club cultures: Music, media, and subcultural capital*: Wesleyan University Press.
